# Corpse Engulfment Generates a Molecular Memory that Primes the Macrophage Inflammatory Response

**DOI:** 10.1016/j.cell.2016.04.049

**Published:** 2016-06-16

**Authors:** Helen Weavers, Iwan R. Evans, Paul Martin, Will Wood

**Affiliations:** 1 Department of Biochemistry, Biomedical Sciences, University of Bristol, Bristol BS8 1TD, UK; 2 Department of Cellular and Molecular Medicine, Biomedical Sciences, University of Bristol, Bristol BS8 1TD, UK; 3 Department of Infection, Immunity and Cardiovascular Disease and the Bateson Centre, University of Sheffield, Sheffield S10 2TN, UK; 4 Department of Physiology, Pharmacology and Neuroscience, Biomedical Sciences, University of Bristol, Bristol BS8 1TD, UK; 5 School of Medicine, Cardiff University, Cardiff CF14 4XN, UK; 6 Lee Kong Chiang School of Medicine, Nanyang Technologicial University, Singapore 636921, Singapore

## Abstract

Macrophages are multifunctional cells that perform diverse roles in health and disease. Emerging evidence has suggested that these innate immune cells might also be capable of developing immunological memory, a trait previously associated with the adaptive system alone. While recent studies have focused on the dramatic macrophage reprogramming that follows infection and protects against secondary microbial attack, can macrophages also develop memory in response to other cues? Here, we show that apoptotic corpse engulfment by *Drosophila* macrophages is an essential primer for their inflammatory response to tissue damage and infection in vivo. Priming is triggered via calcium-induced JNK signaling, which leads to upregulation of the damage receptor Draper, thus providing a molecular memory that allows the cell to rapidly respond to subsequent injury or infection. This remarkable plasticity and capacity for memory places macrophages as key therapeutic targets for treatment of inflammatory disorders.

## Highlights

• Phagocytosis of apoptotic cells primes macrophages for future inflammatory response
• Naive macrophages are insensitive to tissue damage and bacterial infection
• Corpse uptake triggers macrophage calcium bursts that potentiate priming
• Calcium-induced JNK primes macrophages by upregulating the damage receptor Draper

## Graphical Abstract

**Figure:**
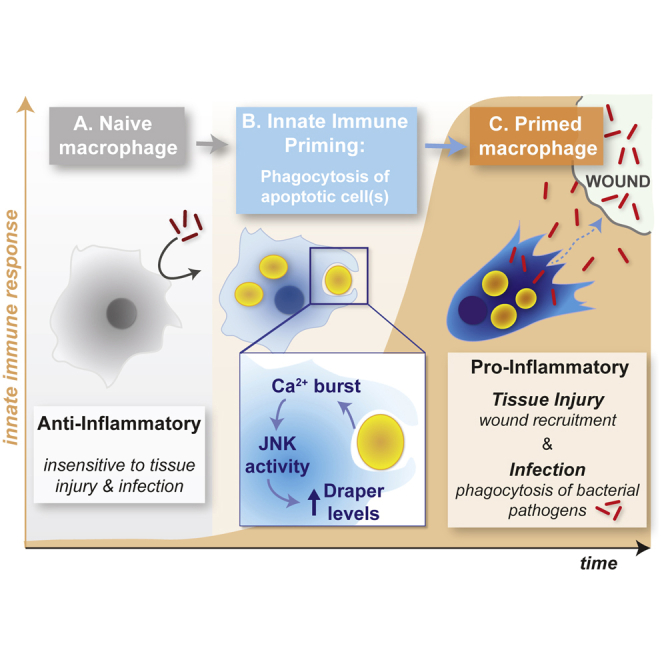

## Teaser

Macrophages that consume apoptotic corpses during fly development become primed for inflammatory responses later in life, establishing a form of molecular memory that aids in the response to bacterial infection and tissue damage.

## Introduction

Traditionally, the innate immune system has been distinguished from the adaptive system by its marked lack of immunological memory (Roitt et al., 2006). While innate (phagocyte-mediated) responses were considered to be the rapid and non-adaptable “first line of defense” against tissue damage and infection, the ability to mount highly specific and adaptable responses had been restricted to the lymphocyte-mediated adaptive system. However, there is now increasing evidence that cells of the innate immune system can become reprogrammed to develop immunological memory of previous encounters (Netea et al., 2011, Quintin et al., 2014).

The development of such innate memory is of clear importance to those organisms that lack an adaptive immune system (such as plants and invertebrates), which can provide valuable resistance to secondary infections in the absence of lymphocyte-mediated responses (Durrant and Dong, 2004, Pham et al., 2007, Rodrigues et al., 2010). However, innate immune memory also provides important protection in mammalian systems, where it functions in parallel with classical B and T cell-dependent adaptive responses. Indeed, mice lacking functional T and B cells can develop cross-protection against secondary bacterial and fungal infections based on innate immune training alone (Kleinnijenhuis et al., 2012, Quintin et al., 2012). Monocytes, macrophages and natural killer (NK) cells have emerged as the main innate immune cells responsible for this priming phenomenon and appear to undergo a profound phenotypic reprogramming upon exposure to microbial stimuli that changes their response to secondary infection (Bowdish et al., 2007).

Until now, research in this field has primarily focused on the innate training that occurs in response to primary infection and the mechanisms by which this confers resistance to secondary microbial attack—a process that has been termed “trained immunity” (Bistoni et al., 1986, Bistoni et al., 1988, Quintin et al., 2012, Vecchiarelli et al., 1989). However, innate immune cells, such as macrophages, are multifunctional cells that not only fight infection, but also perform a range of additional key roles in health and disease. These include the phagocytosis and clearance of dying apoptotic cells, the removal of necrotic cells within damaged tissue, the deposition and remodeling of extracellular matrix (ECM), and the surveillance of abnormal (e.g., cancer) cells (Murray and Wynn, 2011, Wood and Jacinto, 2007). Therefore, it is conceivable that macrophages might also become “trained” and develop immunological memory in response to these other stimuli.

The concept of macrophages as multifunctional cells raises the possibility that exposure to each individual stimulus could reprogram the macrophage so that is responds differently to subsequent stimuli. It is well documented that macrophages display remarkable phenotypic plasticity and can acquire specialized functional phenotypes (often described as M1/M2) in response to a variety of different environmental cytokines and pathogens, giving rise to a spectrum of different macrophage subsets that play diverse roles during host defense, wound repair, and tissue homeostasis (Martinez and Gordon, 2014, Mosser and Edwards, 2008).

One of the key functions of macrophages in vivo is the clearance of dying apoptotic cells, both during normal development/tissue homeostasis (Jacobson et al., 1997, Kerr et al., 1972, Wood et al., 2000) and at sites of inflammation (Martin and Leibovich, 2005). Although apoptosis was traditionally considered to be “immunologically neutral” (Meagher et al., 1992, Stern et al., 1996), more recent studies have suggested it may have powerful immunological effects, being both pro or anti-inflammatory depending on context (Savill et al., 2002).

Determining the exact mechanism by which apoptosis affects macrophage behavior in vivo requires a genetically tractable model in which it is possible to precisely manipulate different macrophage stimuli and intracellular signaling pathways. Here, the *Drosophila* embryo serves as an ideal system, which has been used extensively to model the innate inflammatory response to tissue damage and infection (Evans et al., 2015, Moreira et al., 2010, Razzell et al., 2013, Vlisidou et al., 2009). We exploit the optical translucency of the *Drosophila* embryo to observe macrophage priming in real time in vivo using high-resolution time-lapse imaging.

In this study, we exploit the natural apoptotic cell death that occurs during *Drosophila* development to investigate the role of corpse uptake on the response of macrophages to tissue damage and infection in vivo. We find that corpse phagocytosis is an essential step to prime macrophages for a robust inflammatory recruitment to wounds and uptake of bacteria. We go on to dissect the molecular mechanism by which these immune cells build this memory and show that corpse uptake triggers rapid intracellular calcium bursts within the macrophage, that together with elevated JNK activity and expression of the CED-1 homolog Draper, are required for the macrophage priming effect. Naive macrophages, from *H99* mutants that lack programmed cell death, are unresponsive to wounds and bacterial invasion, but these defects can be rescued by uptake of UV-induced apoptotic corpses or ectopic activation of Draper expression.

We conclude that apoptotic corpses generate a molecular memory within macrophages that has a subsequent pro-inflammatory effect on macrophage behavior that could function in vivo to boost the innate inflammatory response at inflamed sites associated with extensive apoptotic cell death.

## Results

### Macrophages Employ Diverse Strategies to Clear Dying Apoptotic Cells In Vivo

**Figure 1:**
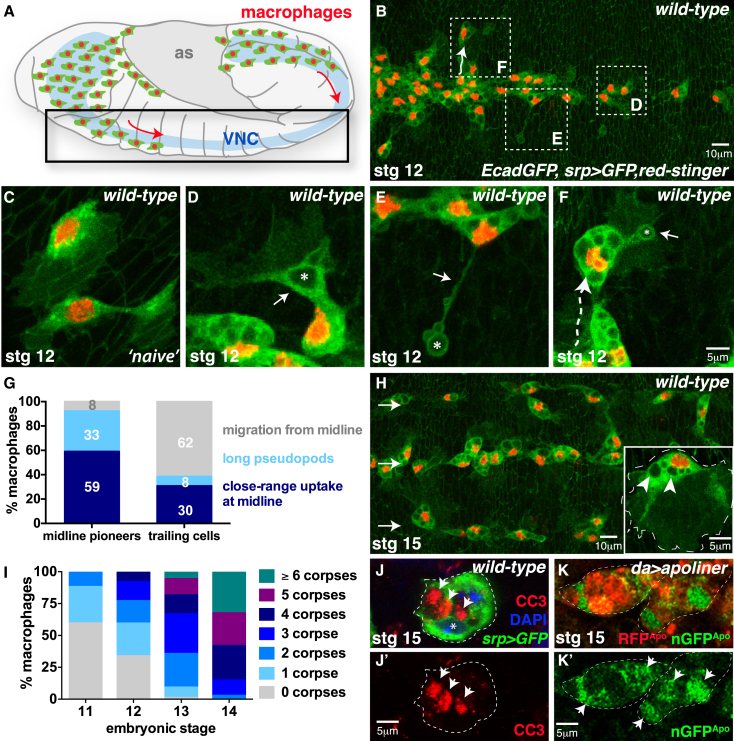
Diverse Macrophage Strategies Clear Dying Apoptotic Cells In Vivo (A–I) *Drosophila* macrophages migrate along the ventral nerve cord (VNC) (arrows, A) and engulf apoptotic cells (B). Naive macrophages (lacking corpses, C) engulf corpses (asterisks) at close range (arrow, D) or using long pseudopods (arrow, E). Trailing macrophages reach outlying corpses (arrow, F) by migration off the midline (dashed line). Uptake strategies are quantified in (G). Corpses accumulate as cytoplasmic vacuoles (arrowheads, inset H; quantified in I). Macrophages reach the three-row arrangement by stage 15 (arrows, H). Macrophages labeled using *srp-Gal4* driving *UAS-red-stinger* (nuclei, red) and *UAS-GFP* (cytoplasm, green). (J–K′) Apoptotic corpses detected in macrophages (green, *srp >GFP*; nuclear DAPI, blue) using cleaved caspase-3 (CC3, red; arrows, J and J′) or the Apoliner caspase sensor (driven ubiquitously by *daughterless-Gal4*; uncleaved Apoliner, red; cleaved nuclear Apoliner, green; arrows, K and K′). See also Movie S1.

During embryogenesis, *Drosophila* macrophages (hemocytes) migrate from their origin in the head mesoderm, along highly stereotypical routes posteriorly along the ventral nerve cord (VNC; Figure 1A) (Tepass et al., 1994). At this time, significant numbers of apoptotic cells are generated during the developmental sculpting of tissues, including neurons within the VNC, and these are rapidly phagocytosed by the migrating macrophages (Movie S1; Figures 1B–1G) (Franc et al., 1999, Suzanne and Steller, 2013, Tepass et al., 1994).

Macrophages initially migrate along the midline of the VNC, guided by local PDGF/VEGF (Pvf) guidance cues expressed along the route of migration (Cho et al., 2002, Wood et al., 2006). The leading “pioneer” cells rarely leave the midline as they migrate posteriorly and predominantly phagocytose apoptotic corpses in their near vicinity (Figure 1D; Movie S1), but occasionally they extend long cytoplasmic arms (“pseudopods”) that contact and engulf outlying apoptotic corpses that are positioned more laterally (up to 40 μm from the midline) (Figure 1E; Movie S1). These pseudopods are rapidly retracted back into the cell body, delivering the apoptotic corpse to the cell for degradation.

In contrast, macrophages positioned further back in the migrating cluster are less spatially constrained (Evans et al., 2010) and migrate laterally out from the midline in response to an apoptotic corpse (arrow, Figure 1B). These macrophages move directly toward the dying cells (Figure 1F; Movie S1), returning back to the midline once engulfment is complete, and only rarely extend the long pseudopods characteristic of the leading cells. The relative contributions of each uptake strategy for the two different populations are depicted in Figure 1G. The differences in uptake strategy most likely reflect early spatial constraints within the developing embryo; macrophages migrate in the extracellular space between the overlying epithelium and underlying VNC, and this space develops in a strict anterior to posterior fashion (Evans et al., 2010).

Individual macrophages progressively phagocytose large numbers of apoptotic corpses that accumulate in the cytoplasm as large vacuoles (inset, Figure 1H). Quantification of corpse uptake reveals that over 80% of macrophages have engulfed and contain at least four corpses by stage 14 (Figure 1I). At this stage, macrophages have completed their developmental migrations and reached the three rows characteristic of mature embryos (Figure 1H). The original populations of “leading” and “trailing” macrophages have become interspersed along the rows. Corpses can be detected by immunostaining for cleaved caspase-3 (Figure 1J). In order to visualize apoptotic cells in living embryos, we expressed the fluorescent caspase sensor Apoliner (Bardet et al., 2008) ubiquitously throughout the embryo (Figure 1K). The Apoliner sensor comprises mRFP and eGFP fluorophores, separated by a specific caspase cleavage site, and is normally retained at the cell surface through a mCD8 transmembrane domain. Upon caspase activation, the sensor is cleaved and eGFP translocates to the nucleus. Using this approach, GFP-positive apoptotic nuclei were observed within macrophages in living embryos (arrowheads, Figure 1K).

### Apoptotic Corpses Prime *Drosophila* Macrophages for Detection of Tissue Damage

**Figure 2:**
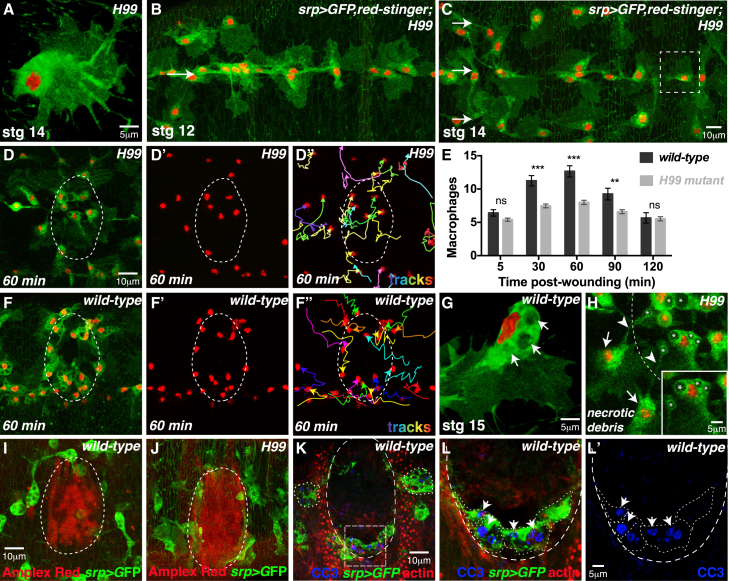
Apoptotic Corpses Prime Macrophages for Detection of Tissue Damage (A–H) *H99* macrophages (*srp-Gal4* driving *red-stinger* and *GFP*) do not encounter corpses (lack of cytoplasmic vacuoles, A) but migrate normally (B) and reach the characteristic three-row arrangement (arrows, C). *H99* macrophages are not robustly recruited to wounds (D–D′′, quantified in E) unlike wild-type macrophages (F–F′′; arrows in G indicate corpses of wild-type macrophage). Data are represented as mean ± SEM; ns, not significant; ^∗∗^p < 0.01 and ^∗∗∗^p < 0.001 via one-way ANOVA followed by Sidak’s multiple comparisons test (E). *H99* macrophages within the wound (dashed line, H) phagocytose necrotic debris (asterisks, H); macrophages outside the wound (arrows, H) extend pseudopods to engulf wound debris (arrowheads, H). (I and J) Wound H_2_O_2_ production (Amplex Red, red) is indistinguishable from wild-type (I) in *H99* mutants (J). Macrophages labeled using *srp >GFP* (green). (K–L′) Apoptotic cells (anti-CC3, blue) are not detected in the wild-type wounded epithelium (K; actin, red). Macrophages outside the wound (dashed outlines, K) and within the wound (dashed outlines, L and L′) contain corpses engulfed earlier during dispersal (arrows, insets L and L′). See also Figure S1 and Movies S2 and S3.

To establish whether apoptotic corpse engulfment influences a macrophage’s ability to respond to tissue damage, we generated embryos that completely lacked apoptosis (Figure 2). We utilized a chromosomal deletion (deficiency *Df(3)H99*) that removes the three genes *head-involution-defective* (*hid*), *reaper* (*rpr*), and *grim* (*grm*) that control developmental programmed cell death in *Drosophila* (Chen et al., 1996, Grether et al., 1995, White et al., 1994, White et al., 1996). In their absence, the normal regimen of programmed cell death does not occur, resulting in embryos that completely lack apoptosis (White et al., 1994). The “naive” macrophages have no opportunity to engulf apoptotic corpses and so lack the large intracellular vacuoles (phagocytosed corpses) characteristic of wild-type cells (cf. Figure 2A with Figure 2G; Movie S2).

**Figure S1:**
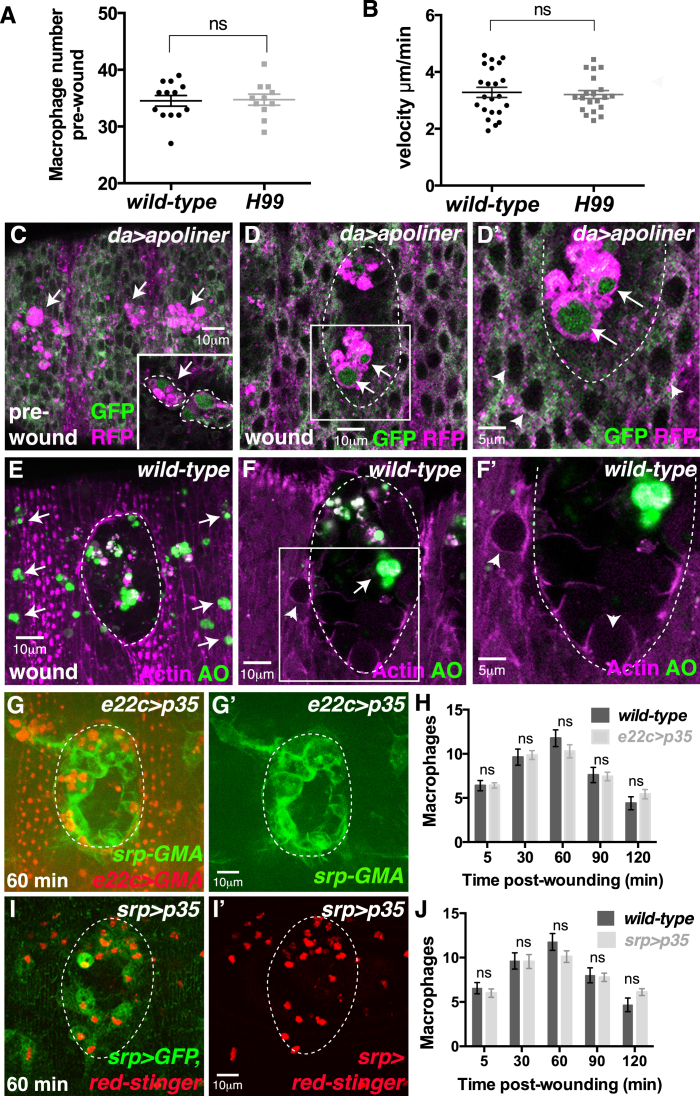
Apoptotic Cell Death Is Not Detected at Wild-Type Wounds, and Apoptotic Caspases Are Not Required in Macrophages for Wound Recruitment, Related to Figure 2 (A and B) The pre-wound *H99* macrophage number (A) and *H99* macrophage developmental migration speed (B) are indistinguishable from wild-type. (C–H) Apoptosis is not detected in the damaged epithelium of wild-type wounds (dashed outline) using the Apoliner caspase sensor (pre-wound in C; wounded, D and inset D′) or Acridine Orange (AO; z-stack projection in E and single section F and inset F′). Apoptosis is not detected in the damaged cells at the wound edge (arrowheads in D′ and F′). Macrophages in unwounded embryos contain apoptotic cells engulfed during prior developmental dispersal (arrows, C and inset; nuclear cleaved Apoliner, green) and these remain following epithelial wounding, observed in macrophages both outside (arrows, E) and inside the wound site (arrows, D and F). Inhibition of apoptotic cell death in the wounded epithelium (red, *e22c-Gal4* driven expression of *UAS-p35*) does not affect macrophage (green, *srp-GMA*) wound recruitment (G and H). (I and J) Macrophage specific expression (using *srp-*Gal4) of the pan-caspase inhibitor *p35* did not affect macrophage wound recruitment (*srp-Gal4 > UAS-GFP, red-*stinger; I-I′ and quantified in J). All data are represented as mean ± SEM; ns, not significant via Mann-Whitney test (A and B) or one-way ANOVA followed by Sidak’s multiple comparisons test (H and J).

Despite the lack of apoptosis in the developing nerve cord (and other tissues), macrophage specification and developmental dispersal along the VNC appears indistinguishable from wild-type (Figure 2B). Macrophages are present in normal numbers (Figure S1A), migrate laterally at speeds similar to wild-type (Figure S1B), and exhibit normal contact inhibition of locomotion (Davis et al., 2012), reaching the stereotypical three-row arrangement by stage 14 (Figure 2C). Apoptotic corpse clearance therefore seems not to be required for early macrophage development or migration (Cho et al., 2002).

However, when *H99* mutant embryos were wounded the normal inflammatory response of macrophages was completely blocked such that naive *H99* macrophages failed to accumulate at the wound site and continued with contact-inhibition migration stereotypical of unwounded embryos (compare Figures 2D–2D′′ with control Figures 2F–2F′′, quantified in Figure 2E; Movie S3), despite normal production of the pro-inflammatory wound attractant H_2_O_2_ (Figures 2I and 2J) (Moreira et al., 2010, Niethammer et al., 2009). Although *H99* macrophages are not attracted over long distances toward the damaged tissue, those macrophages in the immediate vicinity of the wound are seen to phagocytose necrotic wound debris (arrowheads, Figure 2H), indicating that they are not impaired in their ability to detect or phagocytose damaged, dying cells.

Taken together, these data suggest that macrophages generate a molecular memory of their encounter with an apoptotic corpse, and that uptake of these corpses may be an essential pre-requisite for macrophage detection of tissue damage in vivo.

An alternative explanation for the impaired inflammatory recruitment of macrophages in *H99* mutants is that apoptotic corpses at wild-type wounds are a key attractant contributing to macrophage inflammatory chemotaxis. However, this cannot be the case, because apoptosis could not be detected in the damaged epithelium following wounding by CC3 immunostaining (Figures 2K–2L′), Apoliner imaging (Figures S1C–S1D′), or Acridine Orange staining (Figures S1E–S1F′). CC3 positive corpses were observed within macrophages at the wound site (arrowheads, Figures 2L and 2L′), but they are also found outside of the wound (outlines, Figure 2K; also arrows, Figure S1E) and likely reflect corpses engulfed during prior developmental dispersal; indeed 100% of macrophages contain at least one apoptotic corpse in unwounded embryos by this stage (Figures 1H–1K). To further confirm that apoptotic cell death at the wound site does not play a role in macrophage recruitment, we analyzed the inflammatory wound response following inhibition of apoptosis within the wounded epithelium (Figures S1G and S1H). Expression of the pan-caspase inhibitor *p35* (Bump et al., 1995, Hay et al., 1994) throughout the epithelium did not affect macrophage recruitment to wounds (Figures S1G and S1H).

Given that caspases have been implicated in playing a role in cell motility that is unrelated to apoptosis (Geisbrecht and Montell, 2004), we tested whether caspase activity is required within macrophages for their wound recruitment (Figures S1I and S1J). However, macrophage-specific expression of *p35* (Bump et al., 1995, Hay et al., 1994) had no effect on inflammatory wound recruitment, macrophage numbers, or migration speed (Figures S1I and S1J; data not shown).

### Experimental Priming of Naive Macrophages by Apoptotic Corpse Uptake Rescues the Wound Inflammatory Response

**Figure 3:**
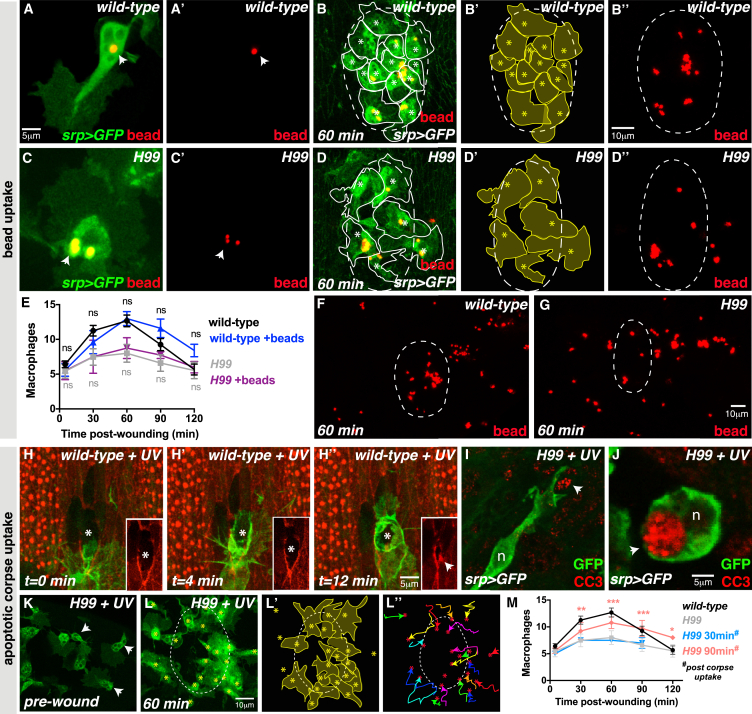
Naive Macrophages Are Experimentally Primed by Corpse Uptake (A–G) Wild-type (A and A′) and *H99* (C and C′) macrophages (*srp >GFP*, green) engulf beads (red; arrows). Wild-type macrophages (yellow outlines, B and B′) with beads (red, high magnification in B′′ and low magnification in F) are robustly recruited to wounds (B–B′′ and E) but *H99* macrophages (yellow outlines, D and D′) with beads (D′′ and low magnification in G) are not (D–D′′ and E). (H–M) UV-induced apoptosis triggered in a single cell (asterisk) that assembles a cortical actin cable and delaminates (arrow) from epithelium (actin, red; inset H–H′′). Macrophage (green, *srp >GFP*) engulfs apoptotic cell (H′ and H′′). UV-triggered corpses detected in *H99* mutants by anti-CC3 (red; arrows) during (I) and after (J) uptake. Corpse uptake by *H99* macrophages (green, *srp >* GFP; pre-wound, K) rescues the wound recruitment defect (macrophages marked by asterisks; outlined in L′) for wounds made 90 min, but not 30 min, post-corpse uptake (L–L′′ and M). For (E) and (M), data are represented as mean ± SEM; ns, not significant; ^∗^p < 0.05, ^∗∗^p < 0.01, and ^∗∗∗^p < 0.001 via one-way ANOVA followed by Sidak’s multiple comparisons test. Significance shown for *H99* 90-min treatment compared to *H99* untreated in (M).

To determine whether macrophages are primed by performing phagocytosis per se or specifically require uptake of apoptotic corpses, we tested whether engulfment of fluorescent beads (of approximately the same size as a corpse) could artificially prime naive *H99* macrophages (Figures 3A–3G). Both wild-type (Figures 3A and 3A′) and naive *H99* macrophages (Figures 3C and 3C′) readily phagocytose fluorescent beads injected into the hemolymph. Bead uptake did not, however, rescue the inflammatory wound recruitment defect of naive *H99* macrophages (Figures 3D–3D′′, 3E, and low magnification in 3G) nor did it affect the recruitment of wild-type macrophages to wounds (Figures 3B–3B′′, 3E, and low magnification in 3F).

These data suggest that macrophage priming by phagocytosis is specific to the uptake of apoptotic corpses. To test this, we attempted to prime naive *H99* macrophages in vivo by stimulating apoptotic corpse uptake (Figures 3H–3M). Apoptosis can be experimentally induced in individual cells in vivo by a focused pulse of 405 nm (UV) laser illumination (Figures 3H–3H′′) (Moreira et al., 2010). Following UV exposure, the targeted epithelial cell is rapidly extruded from the surrounding epithelium by a contractile actin cable (insets, Figures 3H and 3H′) and the cell delaminates basally into the interior of the embryo (inset, Figure 3H′′). The dying cell is rapidly detected by nearby macrophages that engulf the cell as it delaminates from the epithelium (Figures 3H–3H′′).

UV induces apoptosis in *H99* embryos despite the absence of the upstream *rpr*, *hid*, and *grim* genes (Figures 3I and 3J) (White et al., 1994). In this way, apoptotic corpses could be generated and observed within *H99* macrophages by CC3 staining both during (Figure 3I) and after engulfment (Figure 3J). To attempt to rescue macrophage priming, multiple apoptosis events were triggered in *H99* mutants to ensure that the majority of naive *H99* macrophages had engulfed at least one apoptotic corpse (Figure 3K). Strikingly, this approach successfully rescued macrophage recruitment to tissue damage, when the wounds were made 90 min post-corpse induction (Figures 3L–3L′′ and 3M). This rescue was not observed for wounds made only 30 min following corpse uptake (Figure 3M), suggesting that apoptosis-induced macrophage priming requires more than 30 min post-phagocytosis to alter cell behavior.

### Corpse-Associated Calcium Bursts Are Essential for Macrophage Detection of Tissue Damage

**Figure 4:**
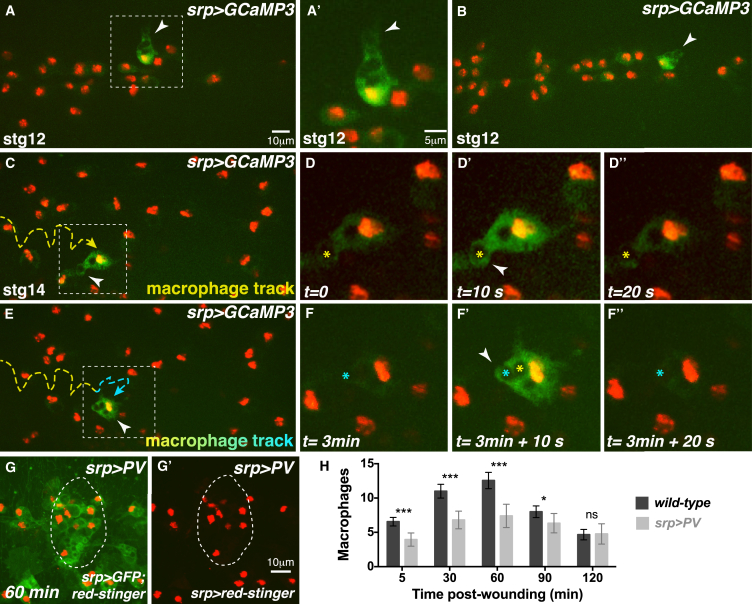
Corpse-Induced Calcium Bursts Prime Macrophages for Wound Recruitment (A–F′′) Wild-type macrophages exhibit rapid calcium flashes (arrowheads; green, *srp-Gal4>UAS-GCaMP3*) upon corpse uptake (A, inset A′ and B). A single calcium flash occurs upon each engulfment (first engulfment in C, insets D–D′′; second engulfment by same cell 3 min later in E, insets F–F′′). Macrophage nuclei (red) labeled using *srp-Gal4 >UAS-red-stinger*. (G and H) Inhibition of calcium bursts (*srp-Gal4>UAS-parvalbumin*) impairs macrophage migration to wounds (G and G′; quantified in H). Macrophages labeled using *srp-Gal4* driven *red-stinger* (nuclei, red) and *GFP* (cytoplasm, green). Data are represented as mean ± SEM; ns, not significant; ^∗^p < 0.05 and ^∗∗∗^p < 0.001 via one-way ANOVA followed by Sidak’s multiple comparisons test (H). See also Figure S2 and Movies S4 and S5.

Intracellular calcium signaling has been linked to apoptotic corpse uptake in worms, flies, and mammals (Cuttell et al., 2008, Gronski et al., 2009, Rubartelli et al., 1997) and was a promising candidate to mediate the macrophage priming response. We monitored the intracellular calcium dynamics of macrophages in real time (Figure 4), by macrophage-specific expression of the intracellular calcium reporter GCaMP3 (Tian et al., 2009). Macrophages experienced frequent but transient increases in cytosolic calcium levels (GCaMP3 fluorescence) that were each associated with apoptotic corpse engulfment (Figures 4A–4F; Movies S4 and S5). We find that 100% of calcium flashes are accompanied by corpse uptake (observed from a total of 68 phagocytic events in 45 different macrophages). Tracking of individual macrophages over time revealed that multiple calcium flashes occur within a single cell (Figures 4C and 4E), with each flash being linked to separate corpse engulfment events (Figures 4D′ and 4F′).

**Figure S2:**
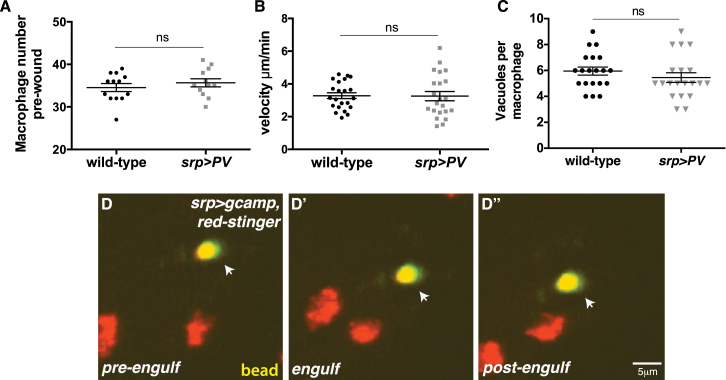
Intracellular Calcium Flashes Are Not Required for Macrophage Specification or Developmental Migration and Are Not Induced by Bead Uptake, Related to Figure 4 (A–C) Inhibition of macrophage calcium signaling (*srp-Gal4* driven expression of *UAS-parvalbumin*) does not affect pre-wound macrophage numbers (A), developmental migration speeds (B) or apoptotic corpse uptake (C) compared to wild-type. (D) Intracellular calcium flashes (*srp-Gal4>UAS-*gcamp3) are not detected during macrophage phagocytosis of fluorescent beads (arrows, yellow). Macrophages labeled using *srp-Gal4* driven expression of *UAS-red-stinger* (red nuclei). Data are represented as mean ± SEM; ns, not significant via Mann-Whitney test (A-C).

To determine whether the macrophage calcium transients were an important mechanism mediating macrophage priming, we expressed *parvalbumin* (*PV*), a vertebrate-specific calcium binding protein that negatively regulates calcium levels in *Drosophila* (Harrisingh et al., 2007, Mortimer et al., 2013), specifically in macrophages (Figure 4G). Inhibition of calcium flashes in macrophages significantly impaired their inflammatory response to tissue damage (Figure 4G and 4G′). There was a dramatic reduction in the number of macrophages recruited to the wound (Figure 4H), similar to that seen in *H99* mutants, although macrophage number, developmental migration speed, and corpse uptake were unaffected (Figures S2A–S2C). These data indicate that apoptotic corpse-associated calcium flashes are indeed required to prime the macrophage response to tissue damage. Notably, phagocytosis of fluorescent beads did not cause an observable increase in GCaMP3 fluorescence (Figures S2D–S2D′′), consistent with our observation that bead uptake is unable to rescue the wound recruitment defect (Figure 3).

### Macrophage Priming Requires Elevated JNK Activity and Draper Expression

**Figure 5:**
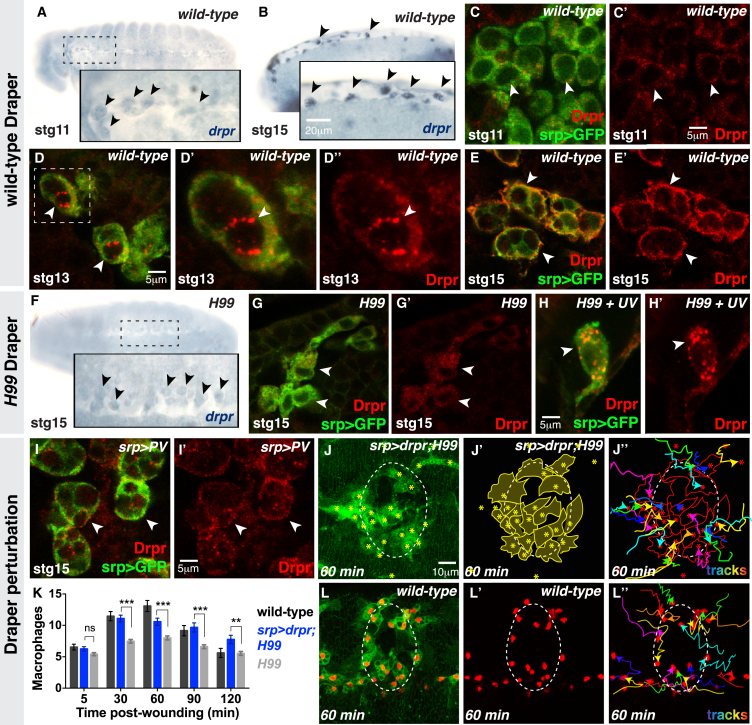
Corpse-Induced Draper Expression Primes Macrophages (A–E′) Draper transcript (A and B) and protein (C and E) levels increase upon corpse uptake. Naive stage 11 macrophages have low levels of Draper transcripts (arrowheads, A) and protein (arrowheads, C and C′) that increase after corpse uptake (D and E); Draper protein relocalizes from corpse-associated punctae (arrowheads, D and D′′) to the cell cortex (arrowheads, E′). (F–L′′) Stage 15 *H99* macrophages have low Draper transcript (arrowheads, F) and protein (arrowheads, G and G′) levels but Draper expression is increased 90 min after UV-induced corpse uptake (arrowheads, H and H′). Inhibition of macrophage calcium signaling also disrupts Draper expression (arrowheads, I). Ectopic Draper expression in *H99* macrophages (driven by *srp-Gal4*) restores macrophage wound recruitment (J–J′′ and K) to wild-type levels (L–L′′). For (K), data are represented as mean ± SEM; ns, not significant; ^∗∗^p < 0.01 and ^∗∗∗^p < 0.001 via one-way ANOVA followed by Sidak’s multiple comparisons test. See also Figure S3.

**Figure S3:**
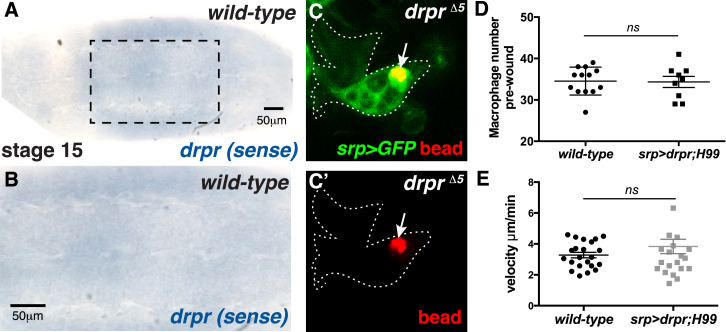
Draper Is Not Required for Bead Uptake, and Draper-Rescued *H99* Mutants Have Normal Macrophage Numbers and Migration Speeds, Related to Figure 5 (A and B) Absence of specific signal using control sense *drpr in situ* probe on wild-type stage 15 embryos. (C and C′) *drpr*^*Δ5*^ mutant macrophages (green, *srp>GFP*) phagocytose beads (red) normally (arrows). (D and E) Ectopic Draper expression in *H99* mutant macrophages does not affect pre-wound macrophage numbers (D) or developmental migration speeds (E). All data are represented as mean ± SEM; ns, not significant via Mann-Whitney test (D and E).

The CED-1 homolog Draper, a phagocytic receptor expressed on macrophages, is required for apoptotic corpse uptake (Manaka et al., 2004) but has also been linked to calcium homeostasis (Cuttell et al., 2008) and macrophage migration to wounds (Evans et al., 2015). Draper might therefore be a pivotal player responsible for apoptotic corpse and calcium-flash induced macrophage priming to tissue damage. Analysis of Draper transcript and protein levels suggests that Draper expression in macrophages is induced following corpse uptake (Figures 5A–5H). In wild-type macrophages, *draper* transcript levels dramatically increase during development following phagocytosis of apoptotic corpses (Figures 5A and 5B; see Figures S3A and S3B for control *sense* staining). We also performed a comprehensive temporal analysis of Draper protein levels in vivo (Figures 5C–5E). Naive stage 11 macrophages exhibit low levels of cytosolic Draper prior to corpse engulfment (Figures 5C and 5C′). Draper expression increases following corpse uptake and Draper protein localizes in punctae around the engulfed corpse in stage 13 macrophages (Figures 5D–5D′′). By stage 15, Draper levels have further increased in mature macrophages and Draper relocalizes to the macrophage cortex (Figures 5E and 5E′).

To test whether Draper expression is induced downstream of corpse uptake, we analyzed Draper levels in *H99* macrophages (Figures 5F–5H). We found only minimal levels of Draper transcript (Figure 5F) and protein (Figures 5G and 5G′) in *H99* macrophages, similar to that observed in naive wild-type macrophages prior to corpse uptake (Figure 5C). However, Draper levels were strongly increased in *H99* macrophages that had engulfed a UV-induced apoptotic corpse 90 min earlier (Figures 5H and 5H′). To determine whether elevated Draper expression also requires corpse-associated calcium flashes, we analyzed Draper levels following inhibition of calcium signaling in macrophages expressing Parvalbumin; Draper levels were low in these macrophages (Figures 5I and 5I′) despite normal corpse uptake (Figure S2C) and were more similar to that observed in naive wild-type (Figure 5C) or *H99* mutant (Figure 5G) macrophages.

Despite the lack of *drpr* expression, *H99* macrophages can efficiently engulf inert beads (Figure 3C) suggesting that Draper expression is not required for bead phagocytosis. Indeed, we find that *drpr*^*Δ5*^-null mutant macrophages engulf beads normally (Figures S3C and S3C′).

These data suggest that the failure of *H99* macrophages to detect wounds might be caused by their lack of corpse-induced Draper expression. We therefore tested whether ectopic expression of Draper within *H99* macrophages could rescue the inflammatory response to tissue damage. Indeed, we found that *H99* macrophages with ectopic Draper expression were now robustly recruited to wounds in a wild-type manner (Figures 5J–5L). Macrophage numbers and developmental migration speeds were unaffected in these embryos (Figures S3D and S3E). Elevated Draper expression therefore appears sufficient to prime macrophages for wound detection, bypassing the need for corpse uptake.

**Figure 6:**
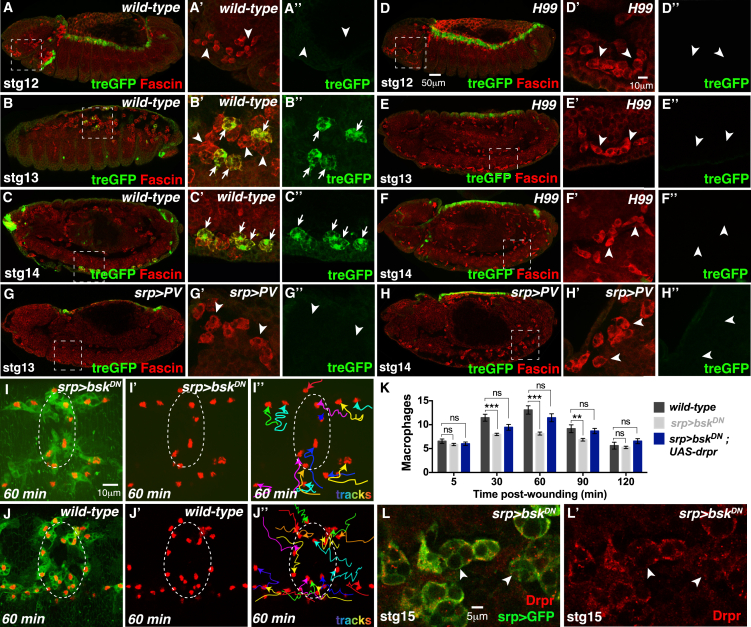
Corpse-Induced JNK Signaling Primes Macrophages (A–H′′) JNK activity (green, *treGFP* reporter) is absent from naive macrophages (red, anti-Fascin) at stage 12 (arrowheads; A–A′′). JNK activity increases as macrophages engulf corpses; JNK activity is initially mosaic (B–B′′) and detected in some macrophages (arrows) but not others (arrowheads) but later spreads to all macrophages (arrows, C–C′′). JNK activity is not detected in naive *H99* macrophages (arrowheads, D–F′′) or following inhibition of calcium signaling (arrowheads, G–H′′). (I–L′) Inhibition of JNK signaling (*srp > bsk*^*DN*^*)* impairs the wound inflammatory response (compare I–I′′ with wild-type in J–J′′; quantified in K) and disrupts Draper expression (red; arrowheads, L and L′), but wound recruitment is rescued by ectopic Draper expression (K). Macrophages were labeled using cytoplasmic GFP (I, J, and L) and nuclear Red-Stinger (I and J). For (K), data are represented as mean ± SEM; ns, not significant; ^∗∗^p < 0.01 and ^∗∗∗^p < 0.001 via one-way ANOVA followed by Sidak’s multiple comparisons test. See also Figure S4.

A recent study has shown that Draper expression and subsequent phagocytic activity within *Drosophila* glial cells is enhanced by JNK signaling (Macdonald et al., 2013). To determine whether JNK signaling in macrophages might be controlling Draper expression levels downstream of corpse uptake, we first examined JNK signaling activity within wild-type macrophages, using the JNK transcriptional reporter *TRE-eGFP* that contains *Drosophila* AP-1 binding sites upstream of the *eGFP* gene (Chatterjee and Bohmann, 2012). *TRE-eGFP* was absent in naive macrophages prior to corpse engulfment (Figures 6A–6A′′), but expression increased during development as macrophages began to clear apoptotic cells (Figures 6B–6B′′ and 6C–6C′′). To assess whether corpse uptake is required for JNK activation, we analyzed *TRE-eGFP* fluorescence in an *H99* mutant background (Figures 6D–6F). *H99* macrophages lacked *TRE-eGFP* reporter activity at all developmental stages (Figures 6D–6F), suggesting JNK signaling is activated downstream of corpse uptake. We also tested whether corpse-associated calcium flashes are required for JNK activation by analyzing the activity of the *TRE-eGFP* reporter following macrophage-specific expression of the calcium inhibitor Parvalbumin (Figures 6G and 6H). Similar to the *H99* mutants, inhibition of calcium signaling completely abrogated *TRE-eGFP* reporter activity within macrophages at all developmental stages examined, suggesting that JNK signaling is activated downstream of macrophage calcium flashes.

**Figure S4:**
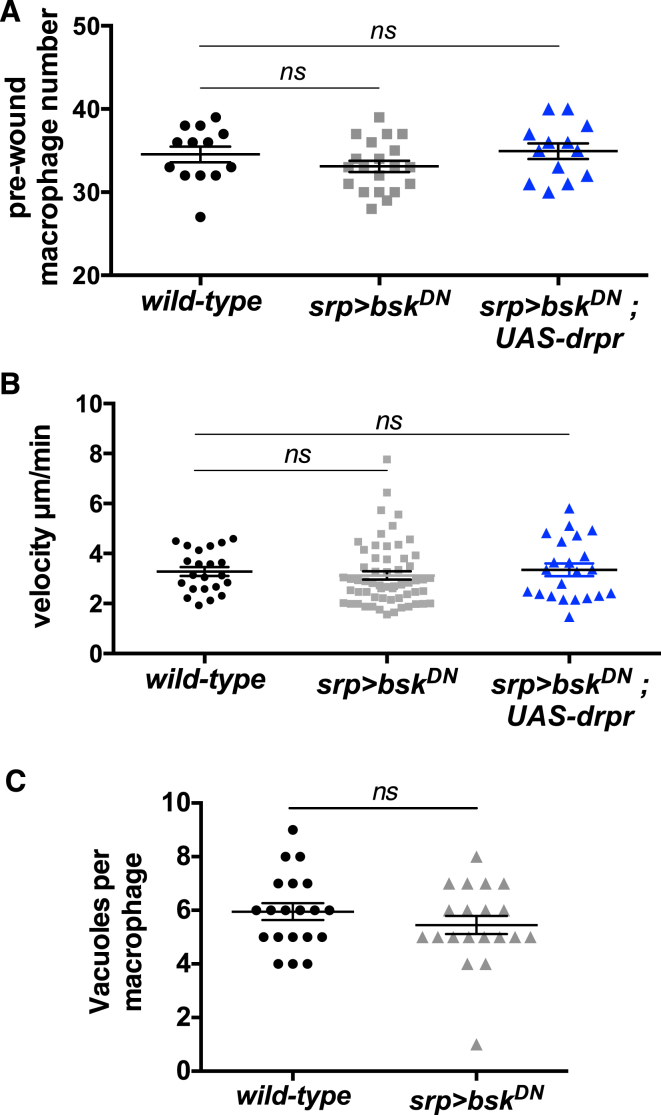
JNK-Inhibited Macrophages Exhibit Normal Pre-wound Numbers, Migration Speeds, and Corpse Uptake, Related to Figure 6 (A–C) Inhibition of JNK signaling in macrophages (*srp-Gal4* driven expression of *UAS-bsk*^*DN*^) does not affect pre-wound macrophage numbers (A), developmental migration speeds (B) or corpse uptake (C). Macrophage numbers and migration speeds are not affected by ectopic expression of Draper in JNK-inhibited macrophages (blue, A and B). All data are represented as mean ± SEM; ns, not significant via Mann-Whitney test (A-C).

We next explored whether macrophages required JNK activity for their inflammatory recruitment to tissue damage. JNK signaling was selectively inhibited in macrophages by expressing a dominant-negative form of Basket (*Drosophila* JNK) (Adachi-Yamada et al., 1999), and this significantly impaired macrophage recruitment to laser-induced wounds (cf. Figures 6I–6I′′ to Figures 6J–6J′′; quantified in 6K). JNK inhibition did not affect macrophage numbers, developmental migration speeds, or corpse uptake (Figures S4A–S4C). Analysis of Draper levels in these macrophages revealed a strong reduction in Draper expression (Figures 6L and 6L′), suggesting that corpse-induced JNK signaling primes the macrophage inflammatory response by activating Draper expression. Consistent with this, we find that overexpression of Draper can rescue the wound recruitment defect caused by macrophage JNK inhibition (Figure 6K).

Given that macrophage priming occurs via JNK signaling and elevated Draper expression, we tested whether ectopically increasing JNK activity or Draper levels in wild-type macrophages could amplify the wound response. However, neither constitutive activation of JNK signaling nor Draper overexpression within wild-type macrophages affected wound recruitment (data not shown).

### Apoptotic Corpse-Associated Calcium Bursts and JNK Signaling also Prime Macrophages for Bacterial Uptake

**Figure 7:**
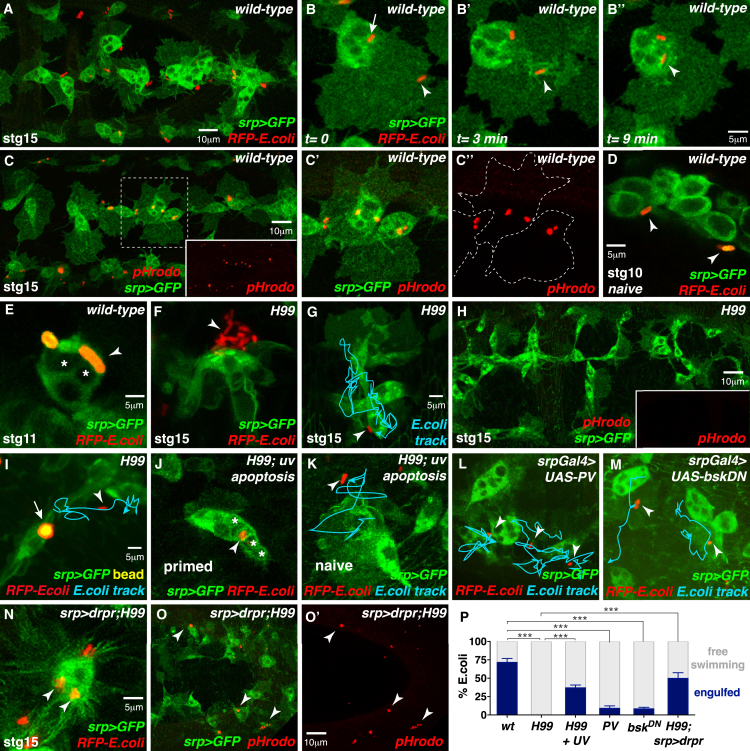
Corpse-Induced Calcium and JNK Signaling also Prime Macrophages for Infection (A–K) Wild-type macrophages (green, *srp >GFP*) engulf *RFP-E. coli* (arrowheads, red; A, insets B–B′′) or pHrodo*-E. coli* (red; C–C′′). Naive stage 10 macrophages do not engulf *RFP-E. coli* (arrowheads, D), but *RFP-E. coli* is taken up by mature stage 11 macrophages (arrowheads, E). *H99* macrophages fail to phagocytose *E. coli* (F and G) or pHrodo-*E. coli* (H); *RFP-E. coli* cluster at the macrophage surface (arrowhead, F) but are not stably bound or engulfed (blue *E. coli* track, G). Bead engulfment (I; arrow, yellow) does not rescue the *H99* bacterial uptake defect (I, blue *E. coli* track; arrowhead, *RFP-E. coli*), but phagocytosis of UV-induced apoptotic corpses (asterisks, J) does rescue uptake (arrowhead, J). *H99* macrophages that lack corpses in the UV-treated embryo fail to engulf *RFP-E. coli* (arrowhead, K; blue, *E. coli* track). (L–P) Inhibition of calcium signaling (L; *srp >parvalbumin*) or JNK activity (M; *srp > bsk*^*DN*^) inhibits macrophage (*srp >* GFP) uptake of *RFP-E. coli* (arrowheads; blue *E. coli* tracks). Ectopic Draper expression in *H99* macrophages (driven by *srp-Gal4*) rescues uptake of *RFP-E. coli* (arrowheads, N) and pHrodo-*E. coli* (arrowheads, O and O′). *E. coli* uptake is quantified in (P) (data are represented as mean ± SEM; ^∗∗∗^p < 0.001 via one-way ANOVA followed by Sidak’s multiple comparisons test). See also Movie S6.

Tissue damage in vivo endangers the host to attack by microbial pathogens, raising the possibility that apoptotic corpse uptake might also prime macrophages to fight infection. *Drosophila* macrophages efficiently recognize and phagocytose bacteria in vivo (Tan et al., 2014, Vlisidou et al., 2009). We monitored macrophage interactions with non-pathogenic *Escherichia coli* (*E. coli*) in real time (Figures 7A; Movie S6). Wild-type macrophages of stage 15 embryos efficiently recognized and bound RFP-expressing *E. coli* at their surface (Figure 7B) that were rapidly phagocytosed into the cell body for degradation (arrowheads, Figures 7B′ and 7B′′). We confirmed that the bacteria had been successfully engulfed by using pH-sensitive pHrodo-*E. coli* that only fluoresce once inside phagosomes (Figures 7C–7C′′). Strikingly, the ability to phagocytose *E. coli* appeared to correlate with macrophage maturity and corpse uptake. Naive macrophages from early (stage 10) embryos, that did not contain any apoptotic corpses, failed to engulf *E. coli* (Figure 7D). However, macrophages from stage 11 embryos that had engulfed apoptotic cells, now also phagocytosed nearby *E. coli* (Figure 7E).

This correlation suggests that, just as for wound recruitment, apoptotic corpse uptake might be a prerequisite for bacterial phagocytosis. We therefore examined whether naive *H99* macrophages are competent to phagocytose *E. coli* (Figures 7F–7H). Following bacterial injection into *H99* mutants, *E. coli* became clustered at the *H99* macrophage surface (Figure 7F) but were not stably bound (*E. coli* motility indicated by blue track, Figure 7G) and were never phagocytosed (Figure 7G; Movie S6; and quantified in Figure 7P). This internalization defect was confirmed by the absence of fluorescence following injection of pHrodo-*E. coli* into *H99* embryos (Figure 7H).

Again, this priming effect does not reflect a general requirement for phagocytosis per se because *H99* macrophages that had engulfed fluorescent beads could not phagocytose *E. coli* (Figure 7I). Importantly, bead uptake itself does not inhibit *E. coli* uptake by wild-type macrophages (data not shown). Just as for the wound priming effect, macrophages are specifically primed for infection by uptake of apoptotic corpses, since *H99* macrophages that had engulfed UV-induced apoptotic corpses were rescued in their ability to phagocytose *E. coli* after 90 min (Figure 7J; quantified in Figure 7P). This rescue was cell autonomous as *H99* macrophages within the same embryo, that had not engulfed a UV-induced corpse, could not engulf *E. coli* (Figure 7K).

To examine whether the same molecular machinery is employed to prime macrophages to detect infection, as demonstrated for tissue damage, we assessed the role of calcium signaling, JNK activity, and Draper levels on macrophage uptake of *E. coli* (Figures 7L–7O). Inhibition of either intracellular calcium bursts (using Parvalbumin) or JNK signaling (using dominant-negative Basket) significantly blocked *E. coli* recognition and phagocytosis (Figures 7L and 7M; quantified in Figure 7P). *E. coli* failed to adhere to the macrophage surface and instead moved freely in the extracellular space evading capture by the macrophages. Ectopic expression of Draper in *H99* macrophages could rescue the uptake of *E. coli* (Figure 7N; quantified in Figure 7P) and pHrodo-*E. coli* (Figures 7O and 7O′) even in the absence of apoptotic corpse engulfment.

## Discussion

Innate immune cells such as macrophages possess remarkable phenotypic plasticity and can become reprogrammed in response to a variety of environmental cytokines and pathogens to develop a type of immunological memory (Martinez and Gordon, 2014, Mosser and Edwards, 2008, Netea et al., 2011). Until now, research has primarily focused on the role of infection in triggering the development of innate immune memory, whereby cells of the innate system become “primed” following primary infection and confer increased resistance to secondary microbial attack (Netea et al., 2011, Quintin et al., 2012, Bistoni et al., 1986, Bistoni et al., 1988, Vecchiarelli et al., 1989). This process has been recently termed “trained immunity.”

However innate cells, particularly macrophages, perform a diverse range of functions during tissue homeostasis and repair, including clearance of apoptotic corpses, tissue remodeling upon wounding, and tumor surveillance (Feng and Martin, 2015, Murray and Wynn, 2011, Noy and Pollard, 2014, Wood and Jacinto, 2007). Yet, the role of these stimuli in macrophage priming has yet to be explored. Here, we demonstrate that macrophages also become reprogrammed in response to phagocytosis of apoptotic corpses, which primes the macrophage for a robust inflammatory response to tissue damage and microbial infection. Using *Drosophila* as our genetically tractable model, we show that naive macrophages, which have not engulfed apoptotic cells (within *H99* mutants that lack programmed cell death), fail to efficiently detect and migrate to sites of sterile tissue damage in vivo*. H99* macrophages also fail to recognize or phagocytose *E. coli* from the extracellular space. Both defects are specifically rescued by uptake of apoptotic corpses by *H99* macrophages but cannot be rescued by phagocytosis per se, as demonstrated by uptake of inert fluorescent beads.

We have dissected the intracellular signals that act downstream of corpse engulfment to elicit these changes in macrophage behavior. We show that apoptotic corpse engulfment rapidly triggers intracellular calcium bursts within the macrophage cytosol (see also Cuttell et al., 2008) and that these are essential for macrophage priming, as genetic abrogation of calcium signaling (using the calcium binding protein Parvalbumin) impaired the macrophage response to tissue damage and bacterial infection.

In recent studies of brain injury, intracellular calcium bursts activated the JNK signaling pathway in injured astrocytes (Gao et al., 2013). In our study, JNK activity was strongly associated with corpse uptake in wild-type macrophages but was absent from both *H99* macrophages (that lacked apoptotic corpses) and Parvalbumin-expressing macrophages (following inhibition of calcium signaling). We show genetically that JNK signaling is required in macrophages for their efficient recruitment to wounds and also for uptake of extracellular *E. coli*.

Activation of JNK signaling in *Drosophila* glial cells enhances phagocytic activity by inducing expression of the phagocytic receptor and CED-1 homolog Draper (Macdonald et al., 2013). Draper, a multi-functional receptor responsible for the phagocytosis of apoptotic cells and invading microbial pathogens (Manaka et al., 2004, Cuttell et al., 2008, Hashimoto et al., 2009), has recently been identified as an important damage receptor controlling macrophage recruitment to sites of tissue damage in vivo (Evans et al., 2015). We therefore postulated that Draper could provide a crucial link between corpse-induced JNK activity and priming for the inflammatory response. We find that levels of Draper transcripts are increased in wild-type macrophages following corpse uptake, and this is accompanied by relocalization of Draper protein from cytosolic punctae to the cell cortex. However, Draper levels are low in naive macrophages of *H99* mutants and following inhibition of calcium or JNK signaling. Furthermore, elevated Draper expression can rescue the wound recruitment and bacterial uptake defect of *H99* mutant macrophages, bypassing the need for apoptotic corpse uptake.

We thus propose a model whereby naive macrophages, prior to corpse uptake, are “anti-inflammatory” and insensitive to tissue injury and infection. We suggest that low basal levels of Draper in naive macrophages are insufficient to allow robust detection of wound-induced tissue damage or invading bacteria. Macrophages can be developmentally reprogrammed, however, by phagocytic uptake of apoptotic corpses, a process that only requires minimal Draper expression. Apoptotic corpse uptake triggers rapid intracellular calcium bursts in the macrophage, which, in turn, promotes JNK activity and increases Draper expression. Primed macrophages display a pro-inflammatory phenotype as the elevated Draper levels sensitize the macrophage for efficient detection of tissue damage and invading bacteria.

We suggest that such corpse-induced macrophage priming confers important protection during host defense in vivo, to augment the innate immune response at sites of inflammation and infection where there are high numbers of dying cells. This is particularly relevant during severe and persistent infections, where apoptotic cell death is a prominent feature of inflamed sites. In the absence of such primed responses, failure to clear the dying apoptotic cells would lead to exacerbated tissue damage as these cells progressed to secondary necrosis. Given that phagocytic clearance of apoptotic corpses has been linked to many inflammatory and autoimmune diseases (Savill et al., 2002, Taylor et al., 2000), further insight into the cellular and molecular mechanisms underlying this priming phenomenon is likely to have important clinical applications.

One of the remaining challenges is to establish the longevity of macrophage priming—whether priming lasts for the remainder of an individual’s lifetime and if this memory is transmitted in the germline. Studies in plants have demonstrated that systemic acquired resistance (SAR)-induced immune priming is transgenerational, as initial infection and induction of SAR in the parental plants conferred resistance to re-infection in their offspring (Luna et al., 2012, Slaughter et al., 2012). Emerging evidence from both plants and animals suggest long-term immune priming or “training” requires large-scale epigenetic reprogramming (Fu and Dong, 2013, Quintin et al., 2014, Slaughter et al., 2012). Whether apoptotic corpses induce such long-term changes in macrophage behavior is an important future challenge.

It is becoming clear that macrophage behavior in vivo is a complex function of all experiences in its immunological past, as each successive stimulus imparts new cellular memory. As we have demonstrated in our study, by changing levels of PAMP and DAMP receptors on their surface, macrophages are able to build a memory of a previous event and consequently adapt and reshape their response to a subsequent assault. The exact macrophage phenotype might also depend on the order in which these encounters occurred, as emerging epidemiological evidence suggests vaccine efficacy could be affected by the order of vaccine administration (Blok et al., 2015). Model organisms such as *Drosophila*, with their advanced genetic tractability and powerful non-invasive live imaging capabilities, will serve as valuable in vivo models to dissect the fundamental cellular and molecular mechanisms responsible for this innate immune priming.

## Experimental Procedures

### *Drosophila* Stocks and Genetics

Fly stocks were maintained according to standard protocols (Greenspan, 1997). All crosses were performed at 25**°**C unless otherwise stated. For a full list of genotypes, see Supplemental Experimental Procedures (Table S1). *Drosophila* mutants and transgenic lines were obtained from the Bloomington Stock Centre unless otherwise stated (Table S1).

### Microscopy and Wounding

Embryos of the appropriate developmental stage were collected from overnight apple juice plates, dechorionated in bleach for 1 min and mounted on double-sided sticky tape on glass slides in 10S Voltalef oil (VWR). Wounds were induced using a nitrogen-pumped Micropoint ablation laser tuned to 435 nm (Andor Technologies) (Razzell et al., 2013). Microinjections and UV-induced apoptosis were performed as before (Tan et al., 2014, Moreira et al., 2010). For Amplex Red staining, dechorionated embryos were incubated in a 1:1 mixture of heptane:Amplex Ultrared solution (50 μM in PBS) for 30 min and mounted as above. Imaging was performed on a PerkinElmer UltraView spinning disc system or Leica TCS SP5 confocal microscope. Image processing was performed using ImageJ (NIH), Adobe Photoshop, or Adobe Illustrator software. For a detailed description of image processing and analysis, see Supplemental Experimental Procedures.

### Immunostaining and In Situ Hybridization

Immunostaining was performed using standard techniques with the antibodies listed (Table S2). An extra amplification step was performed where required using biotinylated secondary antibodies (Vector Laboratories) and streptavidin-conjugated flouorphores (Jackson ImmunoResearch). Carefully staged embryos were oriented and mounted on a glass slide in Vectashield (Vector Labs), and imaging was performed on a Leica SP5 confocal microscope. *drpr* RNA localization was performed by in situ hybridization using Digoxygenin (DIG)-labeled RNA probes generated by in vitro transcription from cDNA templates (GH03529, BDGP). Hybridization and staining was performed according to standard protocols (Nagaso et al., 2001, Tautz and Pfeifle, 1989).

## Author Contributions

H.W. designed and conducted the experiments and wrote the manuscript. I.E. conducted critical preliminary experiments and contributed to experimental design. W.W. and P.M. designed the study, coordinated the project, and helped write the manuscript.

## Supplemental Information

Document S1. Supplemental Experimental Procedures and Tables S1 and S2

Movie S1. Diverse Cellular Strategies Employed by Macrophages to Phagocytose Apoptotic Corpses, Related to Figure 1 Time-lapse movie of the developmental dispersal of Drosophila embryonic macrophages viewed from the ventral aspect. Macrophage nuclei labeled in red (UAS-red-stinger) and cytoplasm in green (UAS-GFP). Macrophages migrate from their origin in the head mesoderm along the ventral midline, gradually dispersing laterally out from the midline in an anterior-posterior manner (top panel). During dispersal, macrophages phagocytose apoptotic corpses generated during developmental tissue sculpting (bottom panel). Macrophages at the midline either clear corpses situated at close-range (bottom, left) or extend long pseudopods to engulf corpses situated more laterally (bottom, centre). Macrophages further back in the migrating cluster, migrate off the midline (‘m’) to phagocytose outlying corpses and return back to the midline once engulfment is complete (bottom, right). Scale bar represents 10μm.

Movie S2. “Naive” Macrophages of H99 Mutants Lack the Cytoplasmic Apoptotic Corpses Characteristic of Wild-Type Macrophages, Related to Figure 2 3D reconstructions of wild-type (left) and H99 mutant (right) macrophages. Macrophage nuclei labeled in red (UAS-red-stinger) and cytoplasm in green (UASGFP). Macrophages in wild-type embryos phagocytose apoptotic corpses generated during developmental tissue morphogenesis and the corpses are visible as large cytoplasmic vacuoles (left). Macrophages from H99 mutants are not exposed to apoptotic corpses and lack the cytoplasmic vacuoles characteristic of wild-type cells (right). Scale bar represents 5 μm.

Movie S3. Naive H99 Macrophages Are Not Recruited to Epithelial Wounds In Vivo, Related to Figure 2 Time-lapse movies of the inflammatory response to laser-induced wounds in the epithelium of wild-type (top panel) and H99 mutants (lower panel). Macrophage nuclei labeled in red (UAS-red-stinger) and cytoplasm in green (UAS-GFP). Wounded region indicated by dashed outline in frame 1 and wound centre indicated by asterisk thereafter. Cell trajectories for a representative subset of macrophages originating outside the wound margin are also shown (right panels). In wild-type, wounding induces a rapid and robust recruitment of macrophages to the damaged tissue (top panels) but naive macrophages in H99 mutants do not accumulate at the wound site and instead continue with normal contact-inhibition of locomotion (lower panels). H99 mutant macrophages already located at the wound margin, do however, phagocytose wound debris (bottom, left). Scale bar represents 15μm.

Movie S4. Macrophages Exhibit Rapid Intracellular Calcium Bursts upon Corpse Engulfment, Related to Figure 4 Time-lapse movie of macrophage calcium signaling during the uptake of apoptotic corpses at stage 12 of development. Macrophage nuclei labeled in red (UAS-red-stinger) and calcium levels in green (UAS-GCaMP3). Rapid and transient rises in cytosolic calcium levels are observed in macrophages upon phagocytic uptake of apoptotic corpses (arrowheads) during early migration down the midline Scale bar represents 10 μm.

Movie S5. Each Apoptotic Engulfment Is Accompanied by a Calcium Flash, Related to Figure 4 Time-lapse movie showing rapid calcium flashes upon corpse engulfment by macrophages in a later stage 13 embryo. On occasion a single macrophage can be seen to flash twice in short succession as it engulfs multiple corpses. Macrophage nuclei labeled in red (UAS-red-stinger) and calcium levels in green (UAS-GCaMP3). Scale bar represents 10 μm.

Movie S6. Wild-type Macrophages, but Not Naive H99 Macrophages, Rapidly Recognize and Engulf *E. coli*, Related to Figure 7 Time-lapse movies of wild-type (left) and H99 mutant (right) macrophage responses to bacterial infection with RFP-tagged E.coli. Macrophage cytoplasm labeled in green (UAS-GFP). Wild-type macrophages efficiently recognise and phagocytose E.coli from the extracellular space (arrowhead, left). However H99 mutant macrophages fail to stably bind and engulf E.coli (arrowhead, right). Scale bar represents 10μm.

Document S2. Article plus Supplemental Information
