# Supplementary material for: Corpse Engulfment Generates a Molecular Memory that Primes the Macrophage Inflammatory Response
Source: Cell. 2016 Jun 16;165(7):1658–71. doi: 10.1016/j.cell.2016.04.049 (PMC4912690; doi:10.1016/j.cell.2016.04.049)
Supplement: Document S1. Supplemental Experimental Procedures and Tables S1 and S2 [file mmc1.pdf]

Cell, Volume *165*

Supplemental Information

Corpse Engulfment Generates a Molecular Memory that Primes the Macrophage Inflammatory Response

Helen Weavers, Iwan R. Evans, Paul Martin, and Will Wood

Extended Experimental Procedures

Drosophila genetics

Details of the precise *Drosophila* stocks used in this study can be found in Table S1.

Image processing and analysis

Z-stacks and time-lapse images were processed and analysed in NIH ImageJ or Volocity (PerkinElmer). Cell tracking was performed in NIH ImageJ (manual tracking) or Volocity (PerkinElmer; automated tracking). Graphical representations and statistical analysis were generated in Prism (GraphPad). For movies of macrophage motility, speed was calculated by dividing the total distance moved over 10 consecutive time-points by the time taken to move that distance. To quantify the macrophage wound response at individual timepoints, the total number of macrophages in contact with, and within the wound margin, were counted from z-stack images.

Immunohistochemistry and *in situ* hybridisation of whole-mount embryos

Embryos were collected from agar apple juice plates, dechorionated in bleach, fixed for 20 minutes in 4% paraformaldehyde, and de-vitellinised in a 1:1 heptane:methanol mixture by vigorous shaking. Wounded embryos were hand-devitellinised using a glass needle. Embryos were washed in 1xPBS/0.1%Triton-X for 3x 10minutes, blocked in 1% BSA in PBS and then incubated in primary antibody overnight at 4°C (see Table S2 for antibody details and dilutions). Primary antibodies were detected using appropriate AlexaFluor488, AlexaFluor568 or AlexFluor647-conjugated secondary antibodies (Molecular Probes). Actin was visualized by incubating in rhodamine-conjugated Phalloidin (Sigma) diluted 1:20 in PBS-TX-BSA for two hours. RNA probes for *drpr in situ* hybridization were made from cDNA obtained from the *Drosophila* Genome Resource Centre (GH03529 clone). For light microscope analysis, embryos were either dehydrated before mounting in Durcupan (Sigma), or alternatively mounted in 80% glycerol to facilitate embryo rolling. For fluorescent analysis, all embryos were mounted in Vectashield (Vector Laboratories) and imaged using a Leica SP5 scanning confocal microscope and LSM software.

Supplemental Tables

Table S1. *Drosophila* Genotypes Used in this Study, Related to Experimental Procedures.

Genotype	Source	Reference
srp-Gal4	Katja Bruckner, UCSF	Brückner et al., 2004
e22c-Gal4	Bloomington Stock Centre	Dierick and Bejsovec, 1998
da-Gal4	Bloomington Stock Centre	Wodarz et al., 1995
drprΔ5	Marc Freeman, UMass	Freeman et al., 2003
UAS-nuclear red stinger	Brian Stramer, KCL	Barolo et al., 2004
UAS-draper-1	Eric Baehrecke, UMass	McPhee et al., 2010
E-cadherin-GFP	Bloomington Stock Centre	Oda et al., 1998
UAS-apoliner	Bloomington Stock Centre	Bardet et al., 2008
Df(3)H99	Bloomington Stock Centre	White et al., 1994
ubi-GFP-Moesin	Bloomington Stock Centre	Dutta et al., 2000
UAS-GCaMP3	Gift from John Gillespie, University of Bristol, UK	Tian et al., 2009
UAS-parvalbumin	Bloomington Stock Centre	Mortimer et al., 2013
tre-GFP	Gift from JP Vincent, NIMR, UK	Chatterjee and Bohmann, 2012
UAS-bskDN	Bloomington Stock Centre	Adachi-Yamada et al., 1999
UAS-p35	Gift from Joaquin de Navascues, Cardiff UK	Hay et al., 1994

Table S2. Antibodies Used in this Study, Related to Experimental Procedures

Antibody	Concentration	Source
Cleaved Caspase-3 #9661	1:50	Cell Signaling
Draper	1:500	Gift from Marc Freeman
GFP	1:500	Abcam
Fascin (sn 7C)	1:100	Developmental Studies Hybridoma Bank (DSHB)

Supplemental References

Barolo, S., Castro, B., and Posakony, J.W. (2004). New Drosophila transgenic reporters: insulated P-element vectors expressing fast-maturing RFP. Biotechniques *36*, 436–440, 442.

Brückner, K., Kockel, L., Duchek, P., Luque, C.M., Rørth, P., and Perrimon, N. (2004). The PDGF/VEGF receptor controls blood cell survival in Drosophila. Dev. Cell *7*, 73–84.

Dierick, H., and Bejsovec, A. (1998). Functional analysis of Wingless reveals a link between intercellular ligand transport and dorsal-cell-specific signaling. Development *125*, 4729–4738.

Dutta, D., Bloor, J.W., Ruiz-Gomez, M., VijayRaghavan, K., and Kiehart, D.P. (2000). Real-time imaging of morphogenetic movements in Drosophila using Gal4-UAS-driven expression of GFP fused to the actin-binding domain of moesin. Genesis *34*, 146–151.

Freeman, M.R., Delrow, J., Kim, J., Johnson, E., and Doe, C.Q. (2003). Unwrapping glial biology: Gcm target genes regulating glial development, diversification, and function. Neuron *38*, 567–580.

McPhee, C.K., Logan, M.A., Freeman, M.R., and Baehrecke, E.H. (2010). Activation of autophagy during cell death requires the engulfment receptor Draper. Nature *465*, 1093–1096.

Oda, H., Tsukita, S., and Takeichi, M. (1998). Dynamic behavior of the cadherin-based cell-cell adhesion system during Drosophila gastrulation. Dev. Biol. *203*, 435–450.

Wodarz, A., Hinz, U., Engelbert, M., and Knust, E. (1995). Expression of crumbs confers apical character on plasma membrane domains of ectodermal epithelia of Drosophila. Cell *82*, 67–76.
