# Supplementary figures and images for: Corpse Engulfment Generates a Molecular Memory that Primes the Macrophage Inflammatory Response
Source: Cell. 2016 Jun 16;165(7):1658–71. doi: 10.1016/j.cell.2016.04.049 (PMC4912690; doi:10.1016/j.cell.2016.04.049)

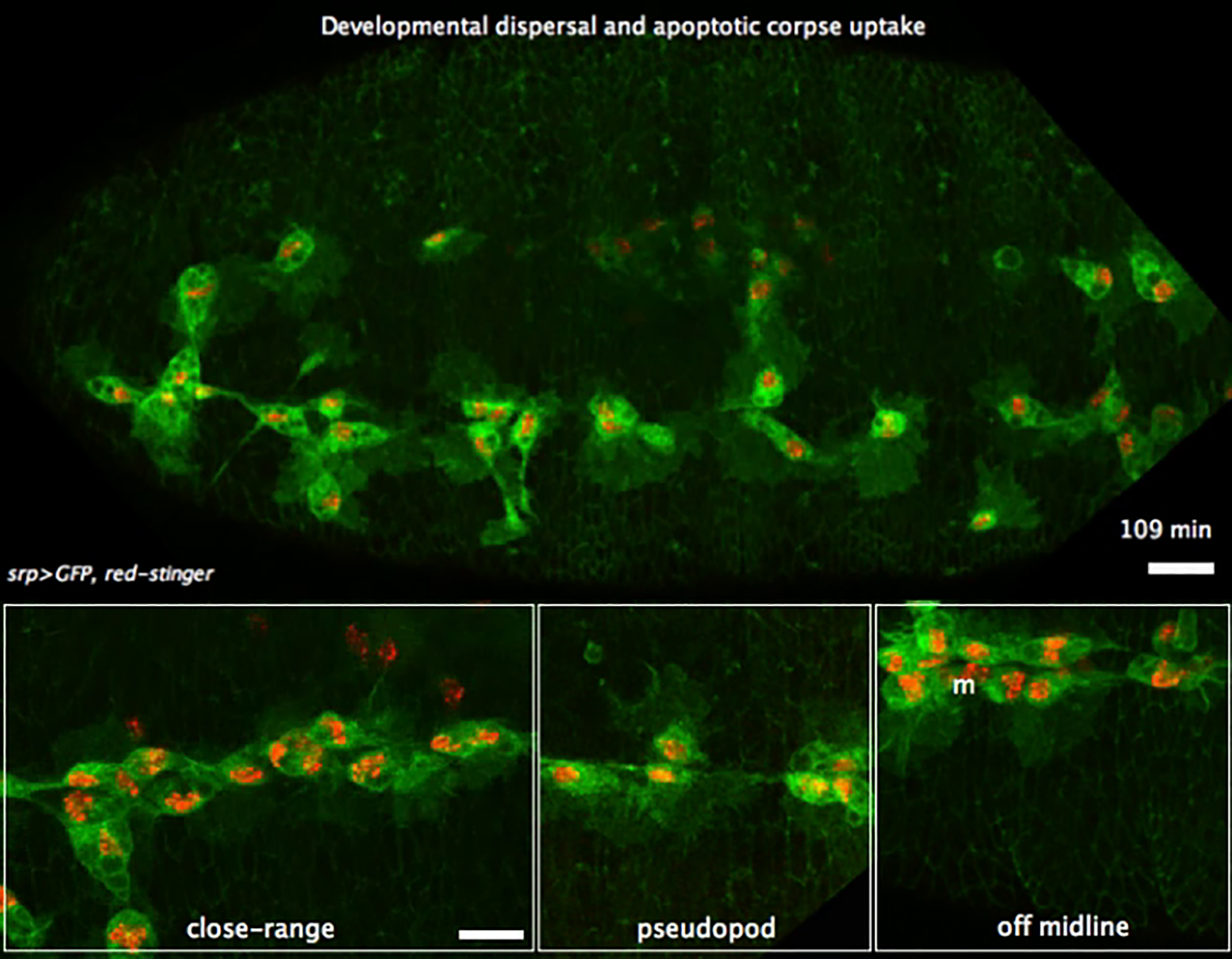

Supplement: Movie S1. Diverse Cellular Strategies Employed by Macrophages to Phagocytose Apoptotic Corpses, Related to Figure 1 — Time-lapse movie of the developmental dispersal of Drosophila embryonic macrophages viewed from the ventral aspect. Macrophage nuclei labeled in red (UAS-red-stinger) and cytoplasm in green (UAS-GFP). Macrophages migrate from their origin in the head mesoderm along the ventral midline, gradually dispersing laterally out from the midline in an anterior-posterior manner (top panel). During dispersal, macrophages phagocytose apoptotic corpses generated during developmental tissue sculpting (bottom panel). Macrophages at the midline either clear corpses situated at close-range (bottom, left) or extend long pseudopods to engulf corpses situated more laterally (bottom, centre). Macrophages further back in the migrating cluster, migrate off the midline (‘m’) to phagocytose outlying corpses and return back to the midline once engulfment is complete (bottom, right). Scale bar represents 10μm. [file mmc2.jpg]

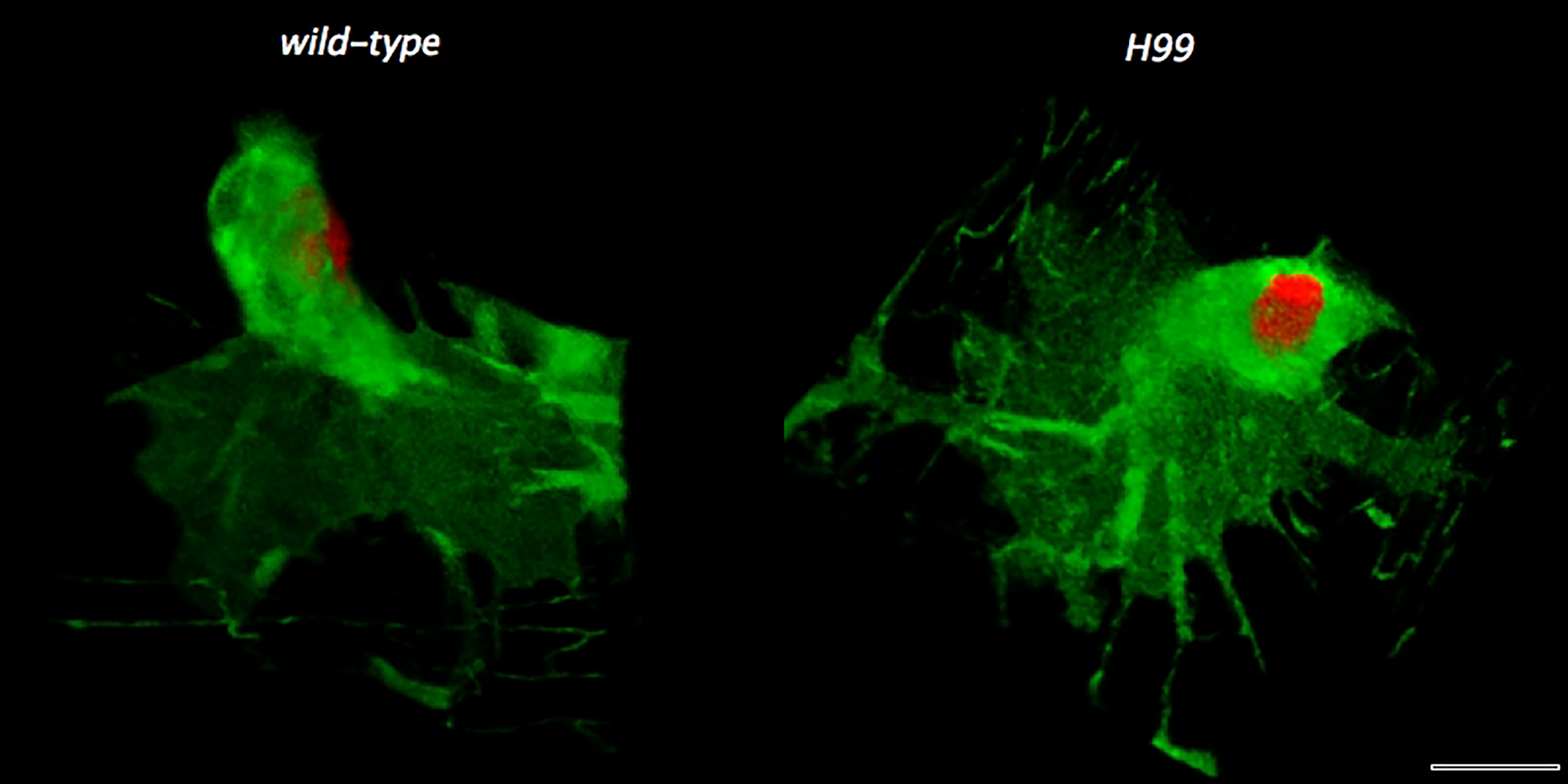

Supplement: Movie S2. “Naive” Macrophages of H99 Mutants Lack the Cytoplasmic Apoptotic Corpses Characteristic of Wild-Type Macrophages, Related to Figure 2 — 3D reconstructions of wild-type (left) and H99 mutant (right) macrophages. Macrophage nuclei labeled in red (UAS-red-stinger) and cytoplasm in green (UASGFP). Macrophages in wild-type embryos phagocytose apoptotic corpses generated during developmental tissue morphogenesis and the corpses are visible as large cytoplasmic vacuoles (left). Macrophages from H99 mutants are not exposed to apoptotic corpses and lack the cytoplasmic vacuoles characteristic of wild-type cells (right). Scale bar represents 5 μm. [file mmc3.jpg]

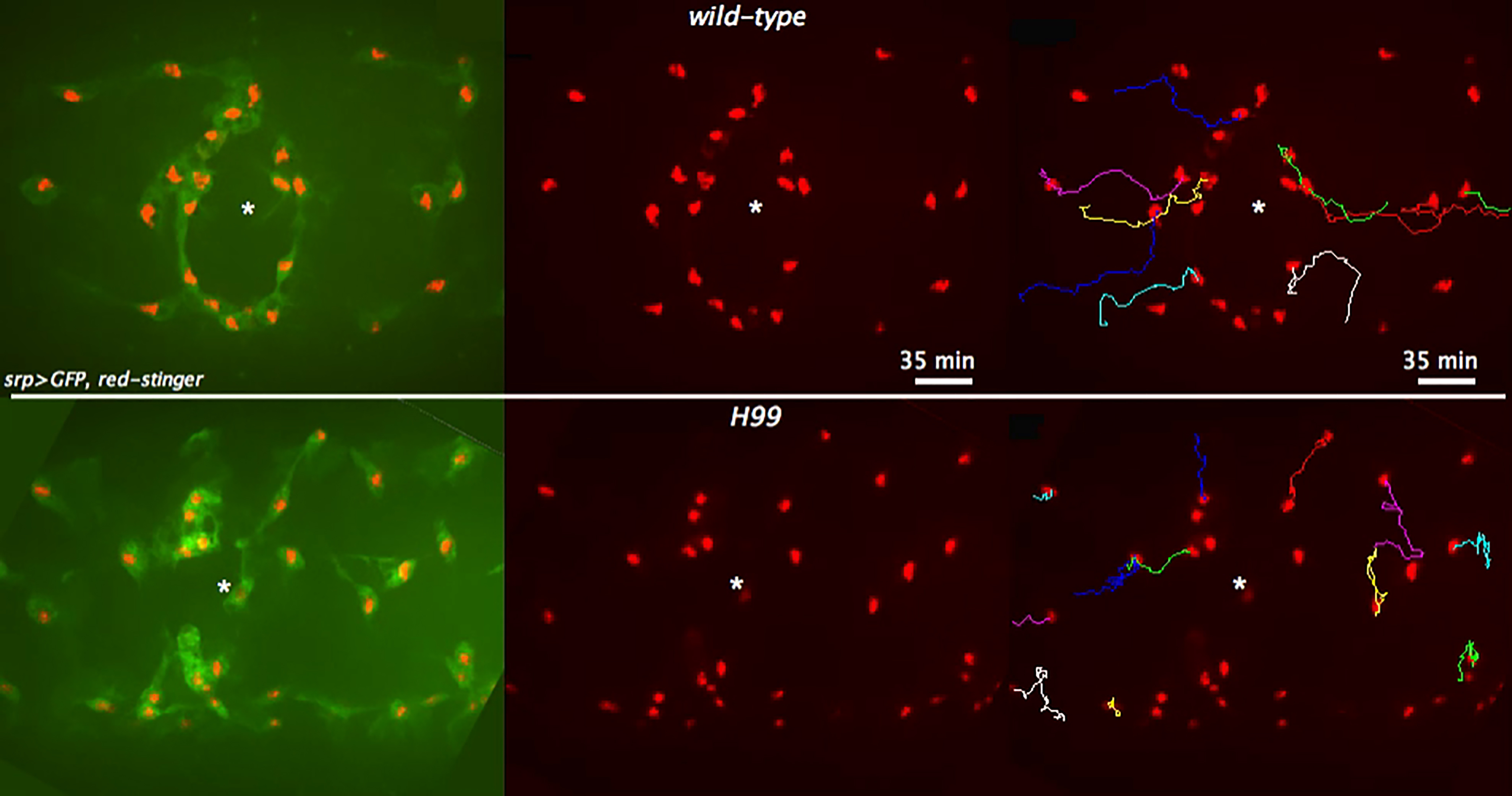

Supplement: Movie S3. Naive H99 Macrophages Are Not Recruited to Epithelial Wounds In Vivo, Related to Figure 2 — Time-lapse movies of the inflammatory response to laser-induced wounds in the epithelium of wild-type (top panel) and H99 mutants (lower panel). Macrophage nuclei labeled in red (UAS-red-stinger) and cytoplasm in green (UAS-GFP). Wounded region indicated by dashed outline in frame 1 and wound centre indicated by asterisk thereafter. Cell trajectories for a representative subset of macrophages originating outside the wound margin are also shown (right panels). In wild-type, wounding induces a rapid and robust recruitment of macrophages to the damaged tissue (top panels) but naive macrophages in H99 mutants do not accumulate at the wound site and instead continue with normal contact-inhibition of locomotion (lower panels). H99 mutant macrophages already located at the wound margin, do however, phagocytose wound debris (bottom, left). Scale bar represents 15μm. [file mmc4.jpg]

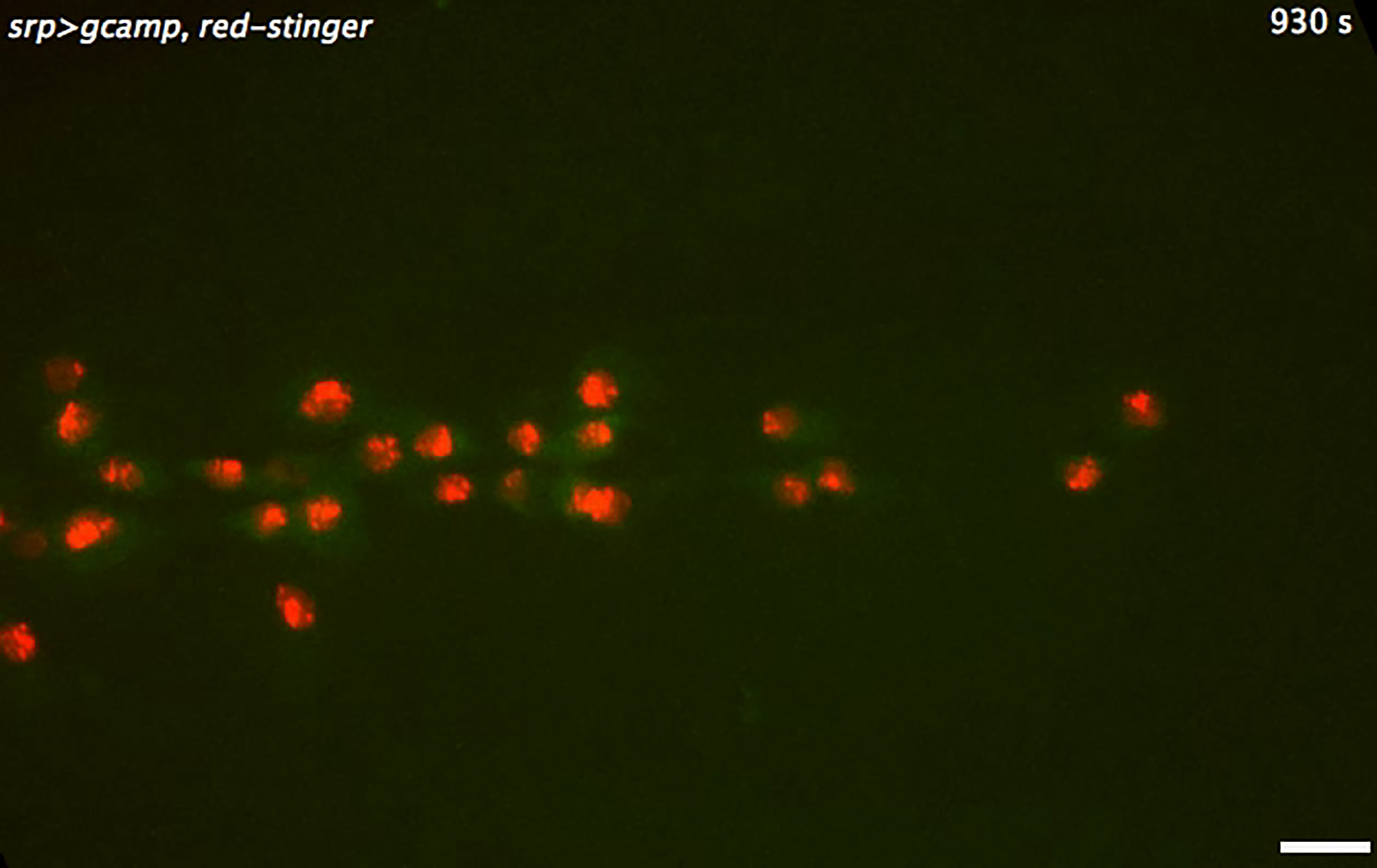

Supplement: Movie S4. Macrophages Exhibit Rapid Intracellular Calcium Bursts upon Corpse Engulfment, Related to Figure 4 — Time-lapse movie of macrophage calcium signaling during the uptake of apoptotic corpses at stage 12 of development. Macrophage nuclei labeled in red (UAS-red-stinger) and calcium levels in green (UAS-GCaMP3). Rapid and transient rises in cytosolic calcium levels are observed in macrophages upon phagocytic uptake of apoptotic corpses (arrowheads) during early migration down the midline Scale bar represents 10 μm. [file mmc5.jpg]

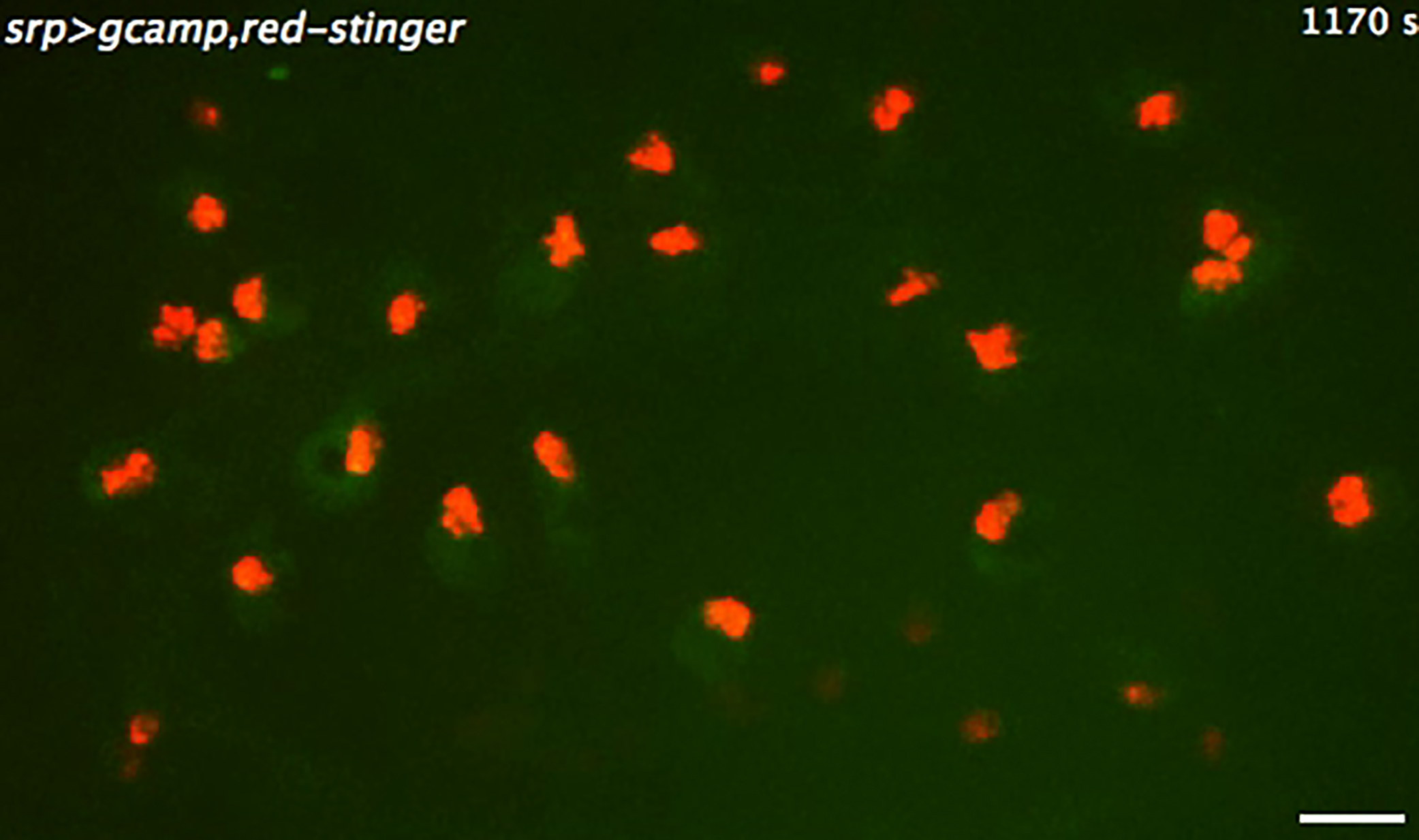

Supplement: Movie S5. Each Apoptotic Engulfment Is Accompanied by a Calcium Flash, Related to Figure 4 — Time-lapse movie showing rapid calcium flashes upon corpse engulfment by macrophages in a later stage 13 embryo. On occasion a single macrophage can be seen to flash twice in short succession as it engulfs multiple corpses. Macrophage nuclei labeled in red (UAS-red-stinger) and calcium levels in green (UAS-GCaMP3). Scale bar represents 10 μm. [file mmc6.jpg]

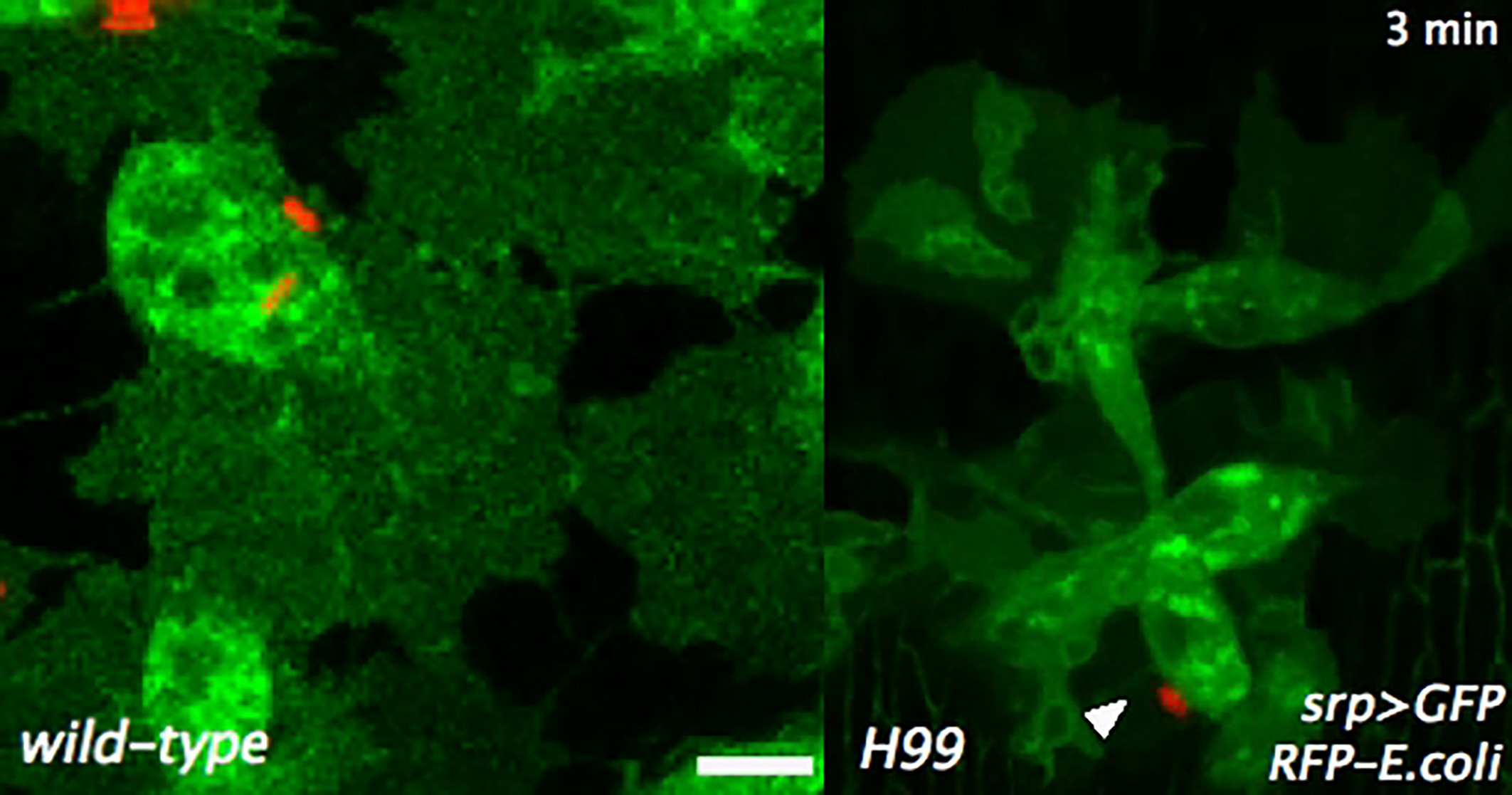

Supplement: Movie S6. Wild-type Macrophages, but Not Naive H99 Macrophages, Rapidly Recognize and Engulf E. coli, Related to Figure 7 — Time-lapse movies of wild-type (left) and H99 mutant (right) macrophage responses to bacterial infection with RFP-tagged E.coli. Macrophage cytoplasm labeled in green (UAS-GFP). Wild-type macrophages efficiently recognise and phagocytose E.coli from the extracellular space (arrowhead, left). However H99 mutant macrophages fail to stably bind and engulf E.coli (arrowhead, right). Scale bar represents 10μm. [file mmc7.jpg]
